# Knowledge and awareness of stroke in the United Arab Emirates: a cross-sectional study of the general population

**DOI:** 10.12688/f1000research.134328.1

**Published:** 2023-09-06

**Authors:** Feras Jirjees, Hala Al-Obaidi, Muna Barakat, Zelal Kharaba, Husam AlSalamat, Zainab Khidhair, Yassen Alfoteih, Eyman Eltayib, Sara Mansour, Souheil Hallit, Diana Malaeb, Hassan Hosseini

**Affiliations:** 1 University of Sharjah, Sharjah, United Arab Emirates; 2 Ajman University, Ajman, United Arab Emirates; 3 Applied Science Private University, Amman, Jordan; 4 Al Ain University, Abu Dhabi, United Arab Emirates; 5 The University of Jordan, Amman, Amman Governorate, Jordan; 6 University of Baghdad, Baghdad, Iraq; 7 City University Ajman, Ajman, United Arab Emirates; 8 Jouf University, Sakaka, Al Jowf, Saudi Arabia; 9 Lebanese International University, Beqaa, Lebanon; 10 Psychiatric Hospital of the Cross, JalEddib, Lebanon; 11 Gulf Medical University, Ajman, United Arab Emirates; 12 Universite Paris-Est Creteil Val de Marne, Créteil, Île-de-France, France

**Keywords:** Stroke, Knowledge, risk factor of stroke, source of information, UAE

## Abstract

Background: The study aimed to assess stroke knowledge among the general population in the United Arab Emirates (UAE) and determine the factors associated with stroke awareness among people.

Methods: A cross-sectional study was conducted in the UAE between September and November 2021. The general population has self-administered an online bilingual survey (Arabic and English) distributed via social media platforms. The questionnaire covered general knowledge about stroke risk factors, consequences of stroke, and responding to somebody with acute stroke attack.

Results: A total of 545 surveys were completed, with more than half of the participants being female (58.5%), married (55.4%) and employed (59.4%). The majority were less than 50 years old (90.5%) and had a university degree (71.0%). Many of the participants (70.8%) had a good general knowledge of stroke; however, around 20% of the participants were able to recognize all symptoms and risk factors of stroke. The most common sources of stroke-related information were the internet/social media (53.6%). Females were better able to correctly identify at least one stroke symptom and outcome than males (p=0.008). University education has significantly affected participants’ ability to identify early stroke symptoms (p=0.001) correctly. In addition, diabetic people were more likely to recognize at least one stroke outcome than non-diabetic people (p=0.039).

Conclusions: The knowledge of stroke was good among highly educated people and females. However, the awareness of all stroke risk factors, symptoms, consequences and risk factors was inadequate among the general population of the UAE. Thus, there is still a gap between recognition of the relevant stroke and taking action among people.

## Introduction

Stroke is a major cause of mortality, morbidity and a leading cause of disability worldwide. The incidence of stroke increases in parallel to age, the prevalence of chronic cardiovascular disease and unhealthy lifestyle. ^1^^,^^2^ Stroke is a health condition that describes a disruption of the blood supply to the brain and thus has devastating health consequences resulting from oxygen starvation and brain cellular damage. ^3^ This damage might lead to paralysis, speech and communication problems, cognitive and memory problems, and death. ^4^

According to World Stroke Organization, stroke has already become a worldwide epidemic and the prevalence rate of stroke reaching around 13.7 million people by the year 2021, and approximately 40% of them are going to die because of the stroke. The data also urged for action as the statistics indicate a rise to 6.7 million expected annual deaths if no actions were taken. ^5^^,^^6^

Prevention is a key to reducing the burden of stroke, with joint efforts required at the individual and community levels. People’s knowledge and stroke awareness are essential for primary stroke prevention and timely access to stroke treatments, including acute reperfusion therapies (such as thrombolysis and mechanical thrombectomy). However, a lack of awareness of stroke among population in many countries has been highlighted. ^7^^–^^10^ Generally, knowledge of risk factors and warning signs in the general population is consistently inadequate or poor. ^11^^,^^12^

The UAE is a fast growing developing country where the last country report on the population number reaching nearly ten million. ^13^ In addition, the UAE is a youth country where 65% of the people are between (25-54 years old). ^14^ There has been about a 40-times increased in country’s population in the past four decades, plus the disparity in education, beliefs, and cultural practices has posed many challenges for shaping population-based public health strategies. ^15^^,^^16^ The increased population has also been associated with an increase prevelance of several non-communicable diseases such as diabetes, asthma, cardiovascular diseases, and cancer. ^17^^–^^19^ However, stroke is considered among the top killer diseases in the country. It is located just behind the ischemic heart diseases and road injuries, where the number of deaths between 1990–2019 has changed by 105%. ^20^

Although there were several calls from national media and practitioners regarding the importance of enhancing awareness among residents in the UAE regarding stroke, only a few available published data have been assessed the awareness level and knowledge among residents in the UAE. ^20^ In a recent study carried out by a group of researchers in one city (the emirate of Sharjah) to assess the knowledge of the stroke among adult residents, data revealed that around 25% of people were misdescribed the disease. ^21^ Another study that evaluated the knowledge of stroke in the Gulf Cooperation Council (GCC) confirmed a poor level of knowledge among GCC communities and urged stroke educational campaigns. ^22^

There are multifactorial aspects of importance in this study. The high prevalence of the disease, the level of stroke knowledge of the public in the country, the level of ignorance of symptoms among the people, and the scarcity of data available for healthcare professionals and health strategists that may help develop programs to increase stroke awareness among the population including rapid response when faced with a case of stroke, which may also help focus on people with poor knowledge of stroke. The current study aimed to assess stroke knowledge among the general population in the UAE and determine the factors associated with stroke awareness.

## Methods

### Ethical approval

The study received ethical approval from the research ethics committee (REC-21-03-20-01) at the University of Sharjah, UAE. All participants agreed to participate in the study by selecting “I agree” on the electronic informed consent form before filling out the questionnaire. All methods were performed in accordance with the relevant guidelines and regulations or declaration of Helsinki.

### Study design and questionnaire

The Strengthening the Reporting of Observational Studies in Epidemiology (STROBE) reporting guideline for cross-sectional studies was adopted and followed as a protocol for conducting this study. ^23^ A cross-sectional observational study was conducted in the UAE between September and November 2021 using an anonymous online survey from the general population. The survey has been developed using the general principles of good survey design. ^24^ An online survey ^25^ was created on Google Forms and distributed via social media applications (WhatsApp and Facebook); through sent it to public general groups in WhatsApp and posted the survey several times in many public pages in Facebook, after obtaining approval from the administrators of these pages. Participation in this study was voluntary, and participants over 18 years old were eligible. Those with a history of stroke were excluded. The survey was self-administered and took approximately ten minutes to complete. The survey was bilingual: Arabic and English. As the Arabic language is the native language of the Emirati citizens and also of the Arabs living in the UAE. The English language survey was conducted for non-Arabic speakers who consist a large ratio of the population in the UAE. A bilingual committee composed of three pharmacy academics translated the English version of the questionnaire into Arabic. An English-speaking translator subsequently performed a back translation and any discrepancies were resolved with the help of the original board. Eight people (authors) and two academic members reviewed the questionnaire and then underwent a five-person pilot test to ensure the clarity of the questions. Then, the questions were modified based on their feedback.

The first section of the questionnaire covered the socio-demographic data. The second section assessed the overall knowledge about stroke and evaluated awareness about stroke risk factors, consequences of stroke, and response when facing somebody with a stroke attack. Moreover, it examined knowledge of early warning signs: participants were awarded one point per correct answer to the above statements. The third section identified sources of information related to stroke among the participants.

### Sample size calculation

The target sample size was estimated to be 384 participants. The number was based on the Raosoft ^®^ software sample size calculator, ^26^ for the minimal sample size needed for an unlimited population size using a confidence interval of 95%, a standard deviation of 0.5 and a margin error of 5%.

### Statistical analysis

The data collected ^27^ were analyzed using the Statistical Package for the Social Sciences (SPSS) version 25.0. Continuous variables were presented as mean ± standard deviation (SD) and 95% confidence interval (CI). Categorical and ordinal variables were presented as frequencies and percentages. Binary logistic regression was performed to identify factors associated with the ability to automatically answer one or more stroke risk factors, one or more warning signs, one or more consequences, and to seek an emergency room once a stroke had developed. Variables with *p* < 0.2 in the bivariate analysis were included in the regression analysis. The results were presented as odds ratios (OR) and 95% CI. Statistical tests were two-tailed and had a statistical significance of *p* < 0.05.

## Results

### Demographic data

A total of 593 surveys were collected from the general population; 48 surveys were excluded because of incomplete responses or refusal to participate. As a result, 545 participants were included in the final study analysis.

More than half of the respondents were females (58.5%), married (55.4%) and employed (59.4%). The majority were less than 50 years (90.5%), non-UAE citizens (73.0%) and held a university bachelor’s degree or above (71.0%). Regarding their medical status, hypertension (12.1%) and dyslipidemia (11.7%) were the most reported comorbid conditions. Moreover, more than half of the respondents (58.3%) stated being only slightly aware of stroke, and one-third (33.8%) knew about stroke from family members or relatives who had this disease before. The demographic characteristics of the respondents are presented in Table 1.

**Table 1.** Participants’ socio-demographic characteristics, past medical history and familiarity with stroke (n = 545).

Variables		Frequency (%)
Socio-demographic characteristics		
Gender	Male	226 (41.5)
	Female	319 (58.5)
Age (years)	18–30	239 (43.9)
	31–50	254 (46.6)
	>50	52 (9.5)
Nationality	UAE	147 (27.0)
	Non-UAE	398 (73.0)
Marital status	Single	229 (42.0)
	Married	302 (55.4)
	Divorced	10 (1.8)
	Widowed	4 (0.7)
Educational level	School level	158 (29.0)
	University level	387 (71.0)
Employment status *	Unemployed	219 (40.6)
	Employed	321 (59.4)
	Very low (<5K)	63 (11.6)
Income level (AED)	Low (5–15K)	166 (30.5)
	Medium (15–25K)	128 (23.5)
	High (>25K)	89 (16.3)
	Prefer not to answer	99 (18.2)
Health insurance	Yes	416 (76.3)
Smoker	Yes	146 (26.8)
Health status	Hypertension	66 (12.1)
	Diabetes Mellitus	38 (7.0)
	Dyslipidemia	64 (11.7)
	Heart diseases	17 (3.1)
	Arrhythmia	18 (3.3)
	Kidney disease	16 (2.9)
	Gastrointestinal diseases	31 (5.7)
	Depression	46 (8.4)
	Migraine	51 (9.4)
	Seizure	6 (1.1)
Familiarity with stroke	History of stroke in the family	81 (14.9)
	Personally know someone with stroke	184 (33.8)

AED, Emirati Dirham (the official currency of the UAE).

* Missing values for employment status (n = 5).

### Stroke knowledge of the participants

**Figure 1.**
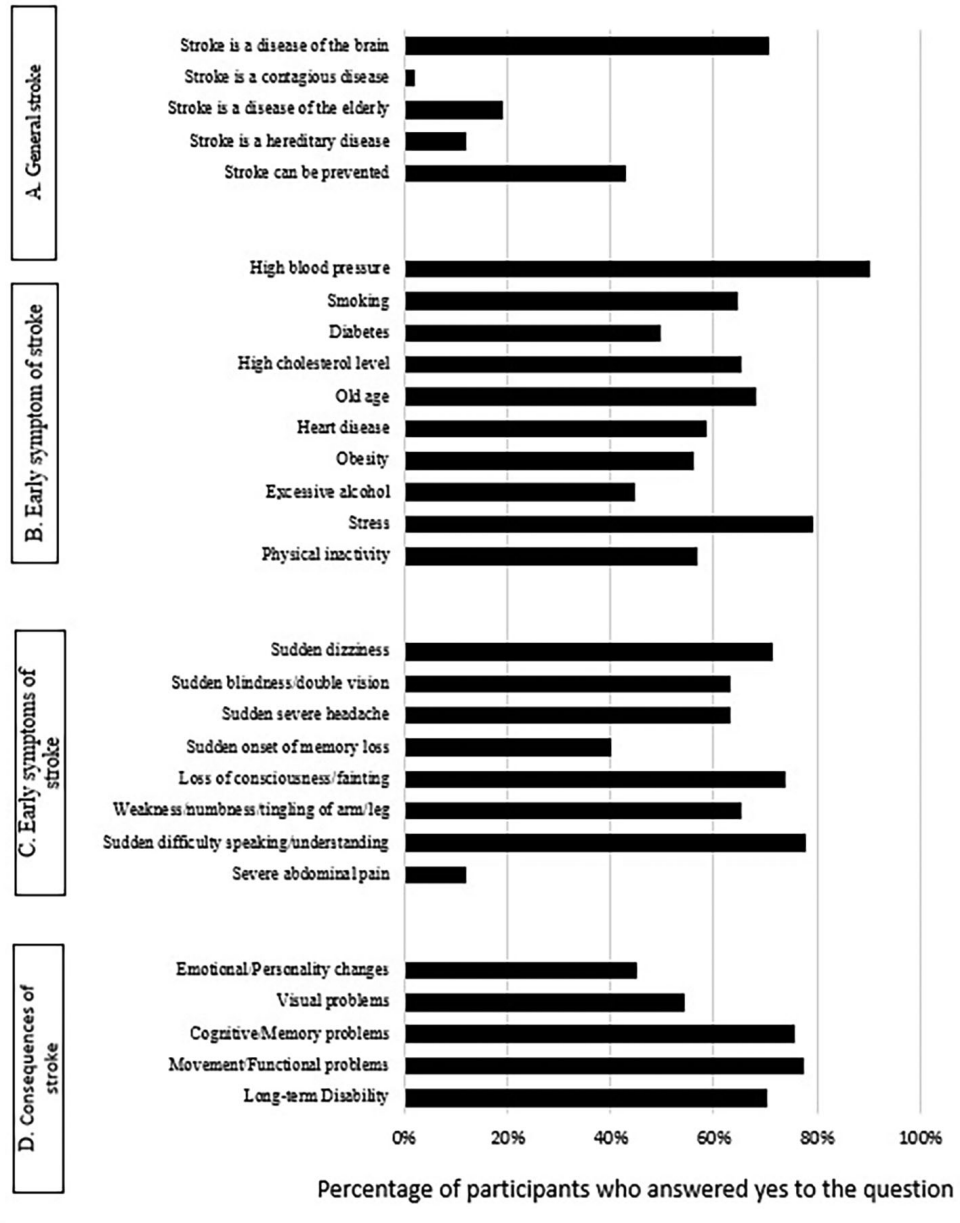
Knowledge of the participants (n = 545) about [A] Stroke, [B] Risk factors related to stroke, [C] Early symptoms of stroke, and [D] Consequences of stroke.

Many respondents (70.8%) recognized the brain as the primary organ affected by a stroke. In comparison, less than half (42.9%) perceived it as a preventable disease ( Figure 1A). As for early stroke symptoms, only 21.3% of participants were able to recognize all symptoms of a stroke. The most often recognized symptoms were sudden difficulty speaking/understanding speech (78.0%), loss of consciousness/fainting (73.8%) and sudden dizziness (71.4%) ( Figure 1B). When asked about possible risk factors, only 22.2% of participants were able to identify all stroke risk factors. The most three frequently identified risk factors were high blood pressure (90.3%), stress (79.3%) and old age (68.1%) ( Figure 1C). Most participants (77.4%) and (75.6%) reported that stroke might lead to functional/movement problem and cognitive/memory problems, respectively. In addition, many participants (70.2%) reported that stroke might lead to long-term disability ( Figure 1D).

Only 21.1% of the respondents believed they do not have good knowledge about stroke and its effects. However, the majority of the participants (82.7%) were curious to have more information related to stroke, including symptoms, emergency signs and responses when facing someone with a stroke attack and the consequences of the stroke. In addition, the majority of participants (94.9%) believed that the role of the family is essential in providing care to a patient with stroke at an early stage. In addition, more than half of the participants (54.7%) believed that stroke disease could make patients’ lives unhappy.

**Figure 2.**
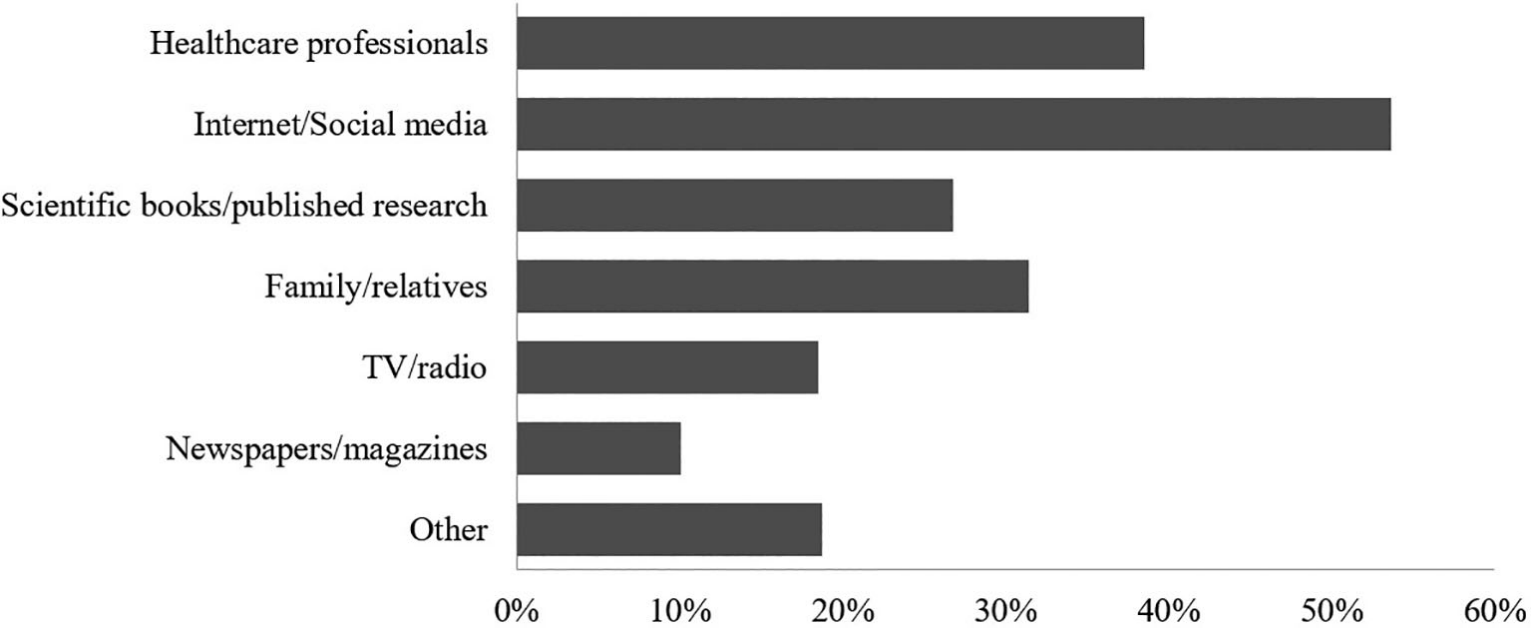
Participants’ Sources of information about stroke.

The most common sources of stroke-related information reported by participants were internet/social media (53.6%), followed by healthcare professionals (38.5%) and family/relatives (31.4%) ( Figure 2).

### Bivariate analysis

In the bivariate analysis, females were better able to significantly identify at least one correct stroke symptom than males (92.8% vs. 85.8%, *p* = 0.008). Moreover, participants with higher levels of education (university level) correctly identified early stroke symptoms compared to those with lower educational levels (92.8% vs. 82.9%, *p* = 0.001), as demonstrated in Table 2.

**Table 2.** Association of risk factors, early symptoms and consequences of stroke with the socio-demographic characteristics and past medical history (n = 545).

Variables		Risk factor(s) identified (≥1)			Early symptom(s) identified (≥1)			Consequence(s) identified (≥1)		
		Yes (n = 544) n (%)	No (n = l) n (%)	P-value	Yes (n = 490) n (%)	No (n = 55) n (%)	*p*-value	Yes (n = 470) n (%)	No (n = 75) n (%)	*p*-value
**Socio-demographic characteristics**										
Gender	Male	226 (100)	0 (0)	1.000	194 (85.8)	32 (14.2)	**0.008**	186 (82.3)	40 (17.7)	**0.025**
	Female	317 (99.7)	1 (0.3)		296 (92.8)	23 (7.2)		284 (89)	35 (11)	
Age (years)	18–30	239 (100)	0 (0)	1.000	216 (90.4)	23 (9.6)	0.729	201 (84.1)	38 (15.9)	0.437
	31–50	253 (99.6)	1 (0.4)		226 (89)	28 (11)		223 (87.8)	31 (12.2)	
	>50	52 (100)	0 (0)		48 (92.3)	4 (7.7)		46 (88.5)	6 (11.5)	
Nationality	UAE	146 (99.3)	1 (0.7)	0.270	127 (86.4)	20 (13.6)	0.098	117 (79.6)	30 (20.4)	**0.006**
	Non-UAE	398 (100)	0 (0)		363 (91.2)	35 (8.8)		353 (88.7)	45 (11.3)	
Marital status	Single	229 (100)	0 (0)	1.000	209 (91.3)	20 (8.7)	0.627	198 (86.5)	31 (13.5)	0.980
	Married	301 (99.7)	1 (0.3)		267 (88.4)	35 (11.6)		259 (85.8)	43 (14.2)	
	Divorced	10 (100)	0 (0)		10 (100)	0 (0)		9 (90)	1 (10)	
	Widowed	4 (100)	0 (0)		4 (100)	0 (0)		4 (100)	0 (0)	
Educational level	School	157 (99.4)	1 (0.6)	0.290	131 (82.9)	27 (17.1)	**0.001**	130 (82.3)	28 (17.7)	0.086
	University	387 (100)	0 (0)		359 (92.8)	28 (7.2)		340 (87.9)	47 (12.1)	
Employment status	Unemployed	218 (99.5)	1 (0.5)	0.406	200 (91.3)	19 (8.7)	0.338	193 (88.1)	26 (11.9)	0.263
	Employed	321 (100)	0 (0)		285 (88.8)	36 (11.2)		272 (84.7)	49 (15.3)	
Income level *	Very Low	63 (100)	0 (0)	1.000	51 (81)	12 (19)	0.079	52 (82.5)	11 (17.5)	0.695
	Low	165 (99.4)	1 (0.6)		152 (91.6)	14 (8.4)		146 (88)	20 (12)	
	Medium	128 (100)	0 (0)		118 (92.2)	10 (7.8)		113 (88.3)	15 (11.7)	
	High	89 (100)	0 (0)		79 (88.8)	10 (11.2)		77 (86.5)	12 (13.5)	
Smoking	No	398 (99.7)	1 (0.3)	1.000	362 (90.7)	37 (9.3)	0.294	349 (87.5)	50 (12.5)	0.168
	Yes	146 (100)	0 (0)		128 (87.7)	18 (12.3)		121 (82.9)	25 (17.1)	
**Health status**										
Hypertension	No	478 (99.8)	1 (0.2)	1.000	431 (90)	48 (10)	0.882	413 (86.2)	66 (13.8)	0.975
	Yes	66 (100)	0 (0)		59 (89.4)	7 (10.6)		57 (86.4)	9 (13.6)	
Diabetes mellitus	No	506 (99.8)	1 (0.2)	1.000	453 (89.3)	54 (10.7)	0.160	433 (85.4)	74 (14.6)	**0.039**
	Yes	38 (100)	0 (0)		37 (97.4)	1 (2.6)		37 (97.4)	1 (2.6)	
Dyslipidemia	No	480 (99.8)	1 (0.2)	1.000	431 (89.6)	50 (10.4)	0.519	416 (86.5)	65 (13.5)	0.645
	Yes	64 (100)	0 (0)		59 (92.2)	5 (7.8)		54 (84.4)	10 (15.6)	
Arrhythmia	No	526 (99.8)	1 (0.2)	1.000	474 (89.9)	53 (10.1)	0.701	453 (86)	74 (14)	0.491
	Yes	16 (88.9)	2 (11.1)		16 (88.9)	2 (11.1)		17 (94.4)	1 (5.6)	
Kidney disease	No	528 (99.8)	1 (0.20	1.000	477 (90.2)	52 (9.8)	0.213	458 (86.6)	71 (13.4)	0.256
	Yes	16 (1000	0 (0)		13 (81.3)	3 (18.8)		12 (75)	4 (25)	
Gastrointestinal disease	No	513 (99.8)	1 (0.2)	1.000	461 (89.7)	53 (10.3)	0.758	440 (85.6)	74 (14.4)	0.104
	Yes	31 (100)	0 (0)		29 (93.5)	2 (6.5)		30 (96.8)	1 (3.2)	
Depression	No	498 (99.5)	1 (0.2)	1.000	447 (89.6)	52 (10.4)	0.608	429 (86)	70 (14)	0.552
	Yes	46 (100)	0 (0)		43 (93.5)	3 (6.5)		41 (89.1)	5 (10.9)	

Numbers in **bold** indicate significant *p*-values ( *p* < 0.05).

^*^ For the variable income level, n = 99 preferred not to answer this question.

Regarding stroke consequences, females compared to males (89% vs. 82.3%, *p* = 0.025) and those with diabetes compared with having no history of diabetes were more likely to recognize at least one consequence of stroke (97.4% vs. 85.4%, *p* = 0.039). Compared to UAE citizens, non-UAE citizen showed significantly higher recognition of stroke consequences (88.7% vs. 79.6%, *p* = 0.006) ( Table 2).

For the response to acute stroke symptoms, none of the studied characteristics were significantly associated with the correct action which is taking patients to the hospital responded to acute stroke symptoms.

### Multivariable logistic regression

When considering the identification of at least an early stroke symptom as the dependent variable, the multivariable analysis showed that females versus males and university compared to school level of education had significantly higher odds (OR of 1.9 and 2.5, respectively). In addition, those with low and medium incomes versus meager incomes were significantly associated with early symptoms identification (OR of 2.5 and 3.1, respectively) ( Table 3).

**Table 3.** Multivariate analysis of associations between socio-demographic data of the participants (n = 545) and identification of risk factors, early symptoms, consequences of stroke and taking a patient to a hospital.

Variables	β (SE)	OR (95% CI)	P-value
**Early symptom(s) identified (≥1)**			
Gender (female vs. male *)	0.661 (0.326)	1.938 (1.023–3.669)	**0.042**
Educational level (university vs. school *)	0.992 (0.328)	2.514 (1.322–4.782)	**0.005**
Diabetes (yes vs. no *)	1.398 (1.041)	4.046 (0.526–31.109)	0.179
**Income level (AED) (versus very low** ^*^ **)**			
Low (5K–15K)	0.930 (0.435)	2.535 (1.080–5.951)	**0.033**
Medium (15K–25K)	1.131 (0.475)	3.099 (1.222–7.858)	**0.017**
High (>2K)	0.683 (0.480)	1.980 (0.772–5.074)	0.155
**Consequence(s) identified (≥1)**			
Gender (female vs. male *)	0.630 (0.255)	1.878 (1.140–3.095)	**0.013**
Nationality (Non-UAE vs. UAE *)	0.723 (0.263)	2.060 (1.230–3.450)	**0.006**
**Taking a patient to a hospital**			
Smoker (yes vs. no *)	0.437 (0.220)	1.548 (1.006–2.381)	**0.047**

β, Beta; SE, standard error; OR. adjusted ratio; CI, confidence interval.

Logistic regression took stroke early symptoms, stroke consequences, and taking a patient who is experiencing a stroke to the hospital as the dependent variables and socio-demographic factors (gender, residence area, educational level, employment status, and smoking history) as independent variables.

Numbers in **bold** indicate significant *p*-values ( *p* < 0.05).

* Reference.

When considering the identification of at least one consequence of stroke as the dependent variable, females versus males and those with non-UAE citizens versus UAE citizens had significantly higher odds (OR of 1.9 and 2.1, respectively) ( Table 3). Regarding the correct response to acute stroke symptoms as the dependent variable, smokers versus non-smokers were more likely to respond by taking the patient to the hospital (OR of 1.5).

## Discussion

A study to assess stroke knowledge among the general UAE population was conducted. More than half of the participants were female and employed. The majority were less than 50 years old and had a university bachelor’s degree or higher. Good general knowledge of stroke was reported in more than two-thirds of the participants. However, just over 20% of the participants recognized all of the symptoms of stroke and were able to identify all of the stroke risk factors.

In 2019, a study by Karkout *et al.* reported an average to low level of knowledge about stroke among the adult population in the UAE. ^21^ Although the majority of our study participants could identify the brain as a typical stroke-affected organ, they were unable to recognize all symptoms and risk factors associated with stroke. However, they identified at least four symptoms, five risk factors, and three-stroke consequences. Visual issues and severe headaches were the least detected signs and symptoms of stroke in the Karkout *et al.* study. ^21^ At the same time, African American race, female gender, and advanced age were the least identified risk factors for stroke. ^21^ Because more than half of the study population identified all information, risk factors, symptoms, and consequences related to stroke, our stroke health literacy outcome measures are higher than similar literature. ^28^^–^^31^

In our study, 99.8% of participants identified at least one risk factor related to stroke. In comparison, previous studies have reported 98.1% among 573 participants in Jordan, ^9^ 85.4% (n = 5391) to 97.8% (n = 551) in Lebanon, ^10^^,^^32^ 76.2% among 822 participants in Australia, ^28^ 76.2% among 609 participants in Iraq, ^33^ 59.6% among 2,884 participants in Spain, ^34^ and 8.6% among 4,671 in Benin of West Africa. ^35^ Conversely, other previous studies have demonstrated poor knowledge of stroke risk factors and symptoms in the general population. ^7^^,^^36^^,^^37^ High blood pressure, hyperglycemia, obesity, renal dysfunction, and hyperlipidemia are all risk factors for stroke, according to the American Heart Association’s latest update for 2021, with a further (47%) attributed to behavioral risk factors like sedentary behavior, smoking, and an unhealthy diet. ^38^ With percentages reaching 50%, hypertension, psychological stress, old age, hypercholesterolemia, smoking, heart disease, physical inactivity, and obesity were the most recognized risk factors for stroke in our study. Unlike a previous Sharjah study in 2019, which found that hypertension (87.0%), hypercholesterolemia (72.6%), obesity (62.3%), and hyperglycemia (59.2%) were the most commonly identified risk factors in the study. ^21^ This demonstrates a more confident tendency among our study participants to recognize stroke risk factors. Furthermore, participants in several studies conducted in Jordan, Saudi Arabia, Lebanon, Iraq, and Morocco reported that hypertension and stress were main risk factors for stroke, at different levels. ^9^^,^^10^^,^^32^^,^^33^^,^^39^^,^^40^ Diabetes Mellitus was somewhat less recognized by our study participants, despite being one of the most frequent modifiable risk factors for stroke (49.7%). This finding has been observed in other investigations in Jordan, Iraq and Morocco. ^9^^,^^33^^,^^40^

With regard to stroke symptoms, participants in our study expressed a high percentage recalling at least one stroke symptom (89.9%) compared to studies in Jordan (95.5%), ^9^ Iraq (76.2%), ^33^ Portugal (74.2%), ^41^ Norway (70.7%), ^42^ Canada (69.5%), ^43^ Oman (68.0%), ^44^ Korea (65%), ^45^ Lebanon (68.2%), ^10^ and Benin of west Africa (4.9%). ^35^ In contrast to our study, only (23%) of participants in a prior 2007 study among GCC countries, including the UAE, identified at least one sign or symptom connected to stroke, which is still a low percentage. ^22^ Sudden difficulty speaking or understanding speech was the most frequently reported stroke symptom in our study (78.0%) with same symptom reported in previous studies in Jordan (92.3%), ^9^ Iraq (88.0%), ^33^ China (65.2%), ^46^ Saudi Arabia (63.8%), ^39^ Ireland (54%), ^11^ and Australia (14.2%). ^28^ However, in Omani (65%) and Nigerian (24.4%) populations, sudden weakness on one side of the body was the most prevalent stroke symptom were reported. ^44^^,^^47^

In terms of their attitudes toward stroke, less than half of the participants in our study (23.9%) were encouraged to go to a hospital as soon as possible after a stroke was detected, with no strong correlation to their socio-demographic characteristics. In a recent large study in China involving 3,051 adults, this phenomenon of paradoxical behavior toward seeking immediate medical help was recognized, with around (25%) of participants who recognized at least one of the stroke symptoms in the stroke action scenario not indicating that they would call an ambulance. ^46^ Nevertheless, previous studies emphasized the need for immediate medical care for stroke patients. ^35^^,^^43^^,^^48^^,^^49^ In Oman, 73% of participants reported they would immediately go to the hospital emergency if they suspected a stroke. ^44^ However, percentages from international studies may vary, with only 47% claiming they would go to a hospital if they were suspicious of a stroke. ^50^ In a previous study in Jordan using the same evaluation measure, participants who were well educated, employed, or diagnosed with diabetes expressed a readiness to take a patient to the hospital as soon as possible if they suspected a stroke. ^9^ Such discrepancies necessitate a more in-depth examination to address knowledge gaps in our study population. It is self-evident that a greater understanding of the implications of a stroke would necessitate timely treatment.

Until 2008, a systematic analysis linked the female gender to a superior overall understanding of stroke risk factors and symptoms. ^36^ In our study, adequate knowledge about risk factors was not attributed significantly to any socio-demographic characteristics, but learning about stroke symptoms was attributed significantly to female gender and advanced education while learning about consequences of stroke was attributed significantly to female gender and nationality of participants. In another study, the male gender was found to be a predictor of increased knowledge. ^47^ Previous research has found no consistent gender correlations in favor of such differences in knowledge of stroke’s risk factors, symptoms, or consequences, so whether there are gender-specific variations in knowledge remains debatable and would require further in-depth causality analyses. ^49^^,^^51^^,^^52^ On the other hand, women are more knowledgeable, show a greater interest in health issues, and spend more time looking for information than men. ^53^

In terms of stroke information resources, the internet and social media were regarded as the most relied on among participants, followed by healthcare professionals and family and/or relatives. However, in studies in Jordan and Iraq, the sequence of the sources of information are similar but the percentages of participants are different. ^9^^,^^34^ This is concerning since publicly available health information on social media may not be evidence-based and is frequently misunderstood by the general public. ^40^^,^^54^^,^^55^

Finally, although the levels of awareness and knowledge related to stroke and risk factors among the people of the UAE were high compared with many studies among people in the region and the world, these levels were still not sufficient. Practically, many of the risk factors associated with stroke are relatively high among the people in the UAE such as smoking, diabetes, cardiovascular diseases, non-healthy lifestyle. ^56^^–^^59^ Therefore, organizational health literacy is needed to target individuals with inadequate personal health knowledge related to stroke among the UAE population through systematic, credible, evidence-based, and accessible health awareness tools. Further research on a national scale could confirm more representative findings of the UAE population.

### Limitations

There are some drawbacks to this study that can be identified. First, the study tool (online survey) requires technical requirements (access to internet and mobile/computer), and reading ability, hence the representation of the population may be compromised. Second, information bias connected to on-demand resource accessibility can jeopardize answer credibility. Third, selection bias associated with the snowball collection technique could be an issue, as there is no guarantee for random selection. Unmeasured variables or responses to variables directly or indirectly connected to stroke could cause residual confounding bias. Furthermore, using an online survey rather than a face-to-face meeting puts the study data’s trustworthiness and authenticity in danger. The online poll includes questions relevant to the UAE and a detailed description of the target population and inclusion criteria in the title and invitation message. Given the COVID-19 pandemic’s restriction measures, such a methodology was the best alternative.

## Conclusions

Although the general UAE population has a high level of personal health literacy when it comes to stroke risk factors, symptoms, and consequences, recognition of all stroke symptoms, risk factors, and consequences were low, and there is still a gap between recognizing a stroke-related event and taking immediate action. Better knowledge of many stroke elements was associated with higher education levels and the female gender.

## Data Availability

Open Science Framework: Underlying data for ‘Knowledge and awareness of stroke in the United Arab Emirates: A cross-sectional study of the general population’, https://www.doi.org/10.17605/OSF.IO/DV6FS. ^27^ This project contains the following underlying data:
- Raw Data.xlsx ^27^ Open Science Framework: Extended data for ‘Knowledge and awareness of stroke in the United Arab Emirates: A cross-sectional study of the general population’, https://www.doi.org/10.17605/OSF.IO/DV6FS. ^25^ This project contains the following extended data:
- Stroke Awareness Questionnaire - UAE.docx ^25^ Open Science Framework: STROBE checklist for ‘Knowledge and awareness of stroke in the United Arab Emirates: A cross-sectional study of the general population’, https://www.doi.org/10.17605/OSF.IO/DV6FS. ^23^ Data are available under the terms of the Creative Commons Attribution 4.0 International license (CC-BY 4.0).
