# Supplementary material for: Knowledge and awareness of stroke in the United Arab Emirates: a cross-sectional study of the general population
Source: F1000Res. 2023 Oct 11;12:1112. Originally published 2023 Sep 6. [Version 2] doi: 10.12688/f1000research.134328.2 (PMC10587663; doi:10.12688/f1000research.134328.2)
Supplement: Peer review history

# Reviewer report: Reviewer response for version 2

**DOI:** 10.5256/f1000research.157179.r214274

**Published:** 2023-10-23

**Authors:** Javedh Shareef

**Affiliations:** 1 Department of Clinical Pharmacy & Pharmacology, RAK College of Pharmacy, RAK Medical & Health Sciences University, Ras Al Khaimah, United Arab Emirates

The author has attended to all my comments which i have addressed in my earlier review of the document. I here by give my consent that this manuscript can be accepted for indexing.

Is the work clearly and accurately presented and does it cite the current literature?

Yes

If applicable, is the statistical analysis and its interpretation appropriate?

Yes

Are all the source data underlying the results available to ensure full reproducibility?

Yes

Is the study design appropriate and is the work technically sound?

Yes

Are the conclusions drawn adequately supported by the results?

Yes

Are sufficient details of methods and analysis provided to allow replication by others?

Yes

Reviewer Expertise:

Clinical Pharmacy Practice

I confirm that I have read this submission and believe that I have an appropriate level of expertise to confirm that it is of an acceptable scientific standard.

# Reviewer report: Reviewer response for version 2

**DOI:** 10.5256/f1000research.157179.r214272

**Published:** 2023-10-20

**Authors:** Doaa Alkhalidi

**Affiliations:** 1 Department of Clinical Pharmacy and Pharmacotherapeutics, Dubai Pharmacy College for Girls, Dubai, United Arab Emirates

The authors have completed the revisions required and the revised version is approved. Congratulations!

Is the work clearly and accurately presented and does it cite the current literature?

Yes

If applicable, is the statistical analysis and its interpretation appropriate?

I cannot comment. A qualified statistician is required.

Are all the source data underlying the results available to ensure full reproducibility?

No source data required

Is the study design appropriate and is the work technically sound?

Yes

Are the conclusions drawn adequately supported by the results?

Partly

Are sufficient details of methods and analysis provided to allow replication by others?

Yes

Reviewer Expertise:

NA

I confirm that I have read this submission and believe that I have an appropriate level of expertise to confirm that it is of an acceptable scientific standard.

# Reviewer report: Reviewer response for version 2

**DOI:** 10.5256/f1000research.157179.r214273

**Published:** 2023-10-19

**Authors:** Hadeer Akram Abdulrazzaq Al-Ani

**Affiliations:** 1 Department of Public Health, School of Medicine, University of California Davis, Davis, CA, USA

Congratulations. Thanks to the study's authors for their excellent achievement in completing the implementation, interpretation, and publication. Regards

Is the work clearly and accurately presented and does it cite the current literature?

Yes

If applicable, is the statistical analysis and its interpretation appropriate?

Yes

Are all the source data underlying the results available to ensure full reproducibility?

Yes

Is the study design appropriate and is the work technically sound?

Yes

Are the conclusions drawn adequately supported by the results?

Yes

Are sufficient details of methods and analysis provided to allow replication by others?

Yes

Reviewer Expertise:

Clinical Pharmacy, Public Health, Pharmacogenetics, and Biostatistics

I confirm that I have read this submission and believe that I have an appropriate level of expertise to confirm that it is of an acceptable scientific standard.

# Reviewer report: Reviewer response for version 1

**DOI:** 10.5256/f1000research.147377.r204543

**Published:** 2023-10-02

**Authors:** Doaa Alkhalidi

**Affiliations:** 1 Department of Clinical Pharmacy and Pharmacotherapeutics, Dubai Pharmacy College for Girls, Dubai, United Arab Emirates

- The study is interesting.
- The title is appropriate.
- The written text needs to be revised to ensure that grammatically correct (some mistakes are there).
- The objectives are clearly stated. 
- In the abstract (results), it has been mentioned that Many of the participants (70.8%) had a good general knowledge of stroke; however, around 20% of the participants were able to recognize all symptoms and risk factors of stroke. How good level of knowledge has been decided. This has to be explained under the methods section, through the scoring system applied in the data analysis. In addition, it needs to be reflected in the results section. 
- It has been mentioned in the "Study Design and Questionnaire" section that those with a history of stroke were excluded. The authors need to clarify how the exclusion took place since it is an online survey. 
- The authors need to explain the validation process of the developed questionnaire.
- How was the reliability of the questionnaire ensured?
- In the same section, the scoring system applied needs further elaboration. What about the incorrect answers? 
- In Table 1, the last variable was about Familiarity with a history of stroke: in the family
  Personally know someone with stroke, what is the difference between each of them?
- In the Results section, " Stroke knowledge of the participants" it has been found that (majority of the participants (82.7%) were curious to have more information related to stroke, including symptoms, emergency signs and responses when facing someone with a stroke attack and the consequences of the stroke), how participants curiosity has been measured through the survey?
- The conclusion is not clearly expressed. It has been mentioned "general UAE population has a high level of personal health literacy" But "stroke risk factors, symptoms, and consequences, recognition of all stroke symptoms, risk factors, and consequences were low" as a reader, it is recognized as a contradiction. The conclusion needs to be expressed more clearly, and some recommendations need to be suggested by the authors based on their findings. 

Is the work clearly and accurately presented and does it cite the current literature?

Yes

If applicable, is the statistical analysis and its interpretation appropriate?

I cannot comment. A qualified statistician is required.

Are all the source data underlying the results available to ensure full reproducibility?

No source data required

Is the study design appropriate and is the work technically sound?

Yes

Are the conclusions drawn adequately supported by the results?

Partly

Are sufficient details of methods and analysis provided to allow replication by others?

Yes

Reviewer Expertise:

Areas of research interest include medication safety, pharmacovigilance, and health services research.

I confirm that I have read this submission and believe that I have an appropriate level of expertise to confirm that it is of an acceptable scientific standard, however I have significant reservations, as outlined above.

# Response

**Published:** 2023-10-06

**Authors:** Feras Jirjees

**Affiliations:** University of Sharjah, United Arab Emirates

Dear Reviewer

 Thank you very much for the valuable comments and suggestions.

**Comment (1) **In the abstract (results), it has been mentioned that Many of the participants (70.8%) had a good general knowledge of stroke; however, around 20% of the participants were able to recognize all symptoms and risk factors of stroke. **How good level of knowledge has been decided**. This has to be explained under the methods section, through the scoring system applied in the data analysis. In addition, it needs to be reflected in the results section. 

**Answer /** It is good comment, we change the statement to be *many of the participants (70.8%) recognized the brain as the primary organ affected by a stroke*.

**Comment (2) **It has been mentioned in the "Study Design and Questionnaire" section that those with a history of stroke were excluded. The authors need to clarify how the exclusion took place since it is an online survey. 

**Answer / **The following statement added to the method section: “ *A question was asked whether the participant had ever had a stroke, and if the participant answered yes, the survey was terminated*”.

**Comment (3) **The authors need to explain the validation process of the developed questionnaire.

**Answer / **the following statement added to the methods section **:** *The questionnaire was structured similarly to a survey developed and validated in a published study among the general population in Jordan in all aspects that covered knowledge of the stroke with the exception of sociodemographic factors such as economic status due to the discrepancy between the two countries (9). The mentioned survey has been developed using the general principles of good survey design (24).”*

**Comment (4) **How was the reliability of the questionnaire ensured?

**Answer / **Sorry for missing this important point, reliability test was conducted before the study began. The following statement added to the methods section: “ *The reliability of the questionnaires was tested among 12 subjects during the pilot study to detect any difficulties in understanding the topic and meaning of the questions. The Cronbach's alpha value for the items in the second section related to assessing comprehensive knowledge about stroke, stroke risk factors, and stroke consequences was (α = 0.73)*”.

**Comment (5) **In the same section, the scoring system applied needs further elaboration. What about the incorrect answers?

**Answer / **If the answer is correct, the participant receives a score of one, and if the answer is incorrect, the participant receives a zero. The statement was mentioned in the method section at the Study design and questionnaire: *“participants were awarded one point per correct answer to *…”. The analysis was performed based on the correct answers. The participants with scores (i.e. high correct knowledge), and this is shown in Figure 1 and Table 2. For incorrect answers, they  did not account in the bivariate and multivariate analysis.

**Comment (6) **In Table 1, the last variable was about Familiarity with a history of stroke: in the family Personally know someone with stroke, what is the difference between each of them?

**Answer / “**History of stroke in the family” means there is a history of stroke in the family, while the “Personally know someone with stroke” means friends or people around them.

**Comment (7) **In the Results section, " Stroke knowledge of the participants" it has been found that (majority of the participants (82.7%) were curious to have more information related to stroke, including symptoms, emergency signs and responses when facing someone with a stroke attack and the consequences of the stroke), how participants curiosity has been measured through the survey?

**Answer /** There was a question in the survey that asked about people’s interest in receiving more information related to stroke, including symptoms, emergency signs and responses when facing someone with a stroke attack and the consequences of the stroke. The importance of this question is to assess the needs of the people in the UAE for awareness program and campaign related the topic of the study as a part of health promotion in the country. A statement added to the method section related to that.

**Comment (8) **The conclusion is not clearly expressed. It has been mentioned "general UAE population has a high level of personal health literacy" But "stroke risk factors, symptoms, and consequences, recognition of all stroke symptoms, risk factors, and consequences were low" as a reader, it is recognized as a contradiction. The conclusion needs to be expressed more clearly, and some recommendations need to be suggested by the authors based on their findings. 

**Answer /**

 Thanks for this comment, a word **moderate** has been added to the conclusion section. Many participants reported correct answers, with more than 50% correct answers on most items and more than 75% correct answers on several questions.

 Best regards

# Reviewer report: Reviewer response for version 1

**DOI:** 10.5256/f1000research.147377.r204544

**Published:** 2023-09-28

**Authors:** Hadeer Akram Abdulrazzaq Al-Ani

**Affiliations:** 1 Department of Public Health, School of Medicine, University of California Davis, Davis, CA, USA

I want to congratulate the authors of this study for their excellent work and achievement in implementing and publishing the present study. I have several points that are required to be edited and corrected.

1. Several sentences are required to be rephrased to be clear to the readers, especially in the abstract.
2. In methodology, please write about the questionnaire's validity procedure and reliability outcomes.
3. In the statistical analysis (Methodology ): What do you mean by “Variables with p < 0.2 in the bivariate analysis”? Why did you select the cutoff equal to 0.2? Please justify and support the sentence with a reference.
4. Figure 1 is blurred. Please try to find a clear image.
5. Please be consistent with the background of Table 2.
6. Table 3, please add columns of the df and confidence interval. The readers like to see the validity of outcomes.
7. In Table 3, please correct the level of high income (High (>2K)). Also, justify why the income increase of the early symptoms identified is not significant.
8. In the Discussion, please be consistent in writing the following sentence: “In comparison, previous studies have reported 98.1% among 573 participants in Jordan, 85.4% (n = 5391) to 97.8% (n = 551) in Lebanon, 76.2% among 822 participants in Australia,  76.2% among 609 participants in Iraq, 8.6% among 4,671 in Benin of  West Africa”.
9. Please add future work.

Is the work clearly and accurately presented and does it cite the current literature?

Yes

If applicable, is the statistical analysis and its interpretation appropriate?

Yes

Are all the source data underlying the results available to ensure full reproducibility?

Yes

Is the study design appropriate and is the work technically sound?

Yes

Are the conclusions drawn adequately supported by the results?

Yes

Are sufficient details of methods and analysis provided to allow replication by others?

Yes

Reviewer Expertise:

Clinical Pharmacy, Public Health, Pharmacogenetics, and Biostatistics

I confirm that I have read this submission and believe that I have an appropriate level of expertise to confirm that it is of an acceptable scientific standard, however I have significant reservations, as outlined above.

# Response

**Published:** 2023-10-06

**Authors:** Feras Jirjees

**Affiliations:** University of Sharjah, United Arab Emirates

Dear Reviewer

 I greatly appreciate your valuable feedback and suggestions.

**Comment (1)** Several sentences are required to be rephrased to be clear to the readers, especially in the abstract.

**Answer / **Some corrections have been made in the manuscript which is heighted in track changes mode.

**Comment (2)** In methodology, please write about the questionnaire's validity procedure and reliability outcomes.

**Answer /** The following statement added to the methods section **:** *The questionnaire was structured similarly to a survey developed and validated in a published study among the general population in Jordan in all aspects that covered knowledge of the stroke with the exception of sociodemographic factors such as economic status due to the discrepancy between the two countries (9). The mentioned survey has been developed using the general principles of good survey design (24)”.* In addition, reliability test was conducted before the study began. The following statement added to the methods section: “ *The reliability of the questionnaires was tested among 12 subjects during the pilot study to detect any difficulties in understanding the topic and meaning of the questions. The Cronbach's alpha value for the items in the second section related to assessing comprehensive knowledge about stroke, stroke risk factors, and stroke consequences was (α = 0.73)*”.

**Comment (3)** In the statistical analysis (Methodology ): What do you mean by “Variables with p < 0.2 in the bivariate analysis”? Why did you select the cutoff equal to 0.2? Please justify and support the sentence with a reference.

**Answer / **A justification and support the sentence was added to the methods section.

**Comment (4)** Figure 1 is blurred. Please try to find a clear image.

**Answer / **A better figure is provided to replace the current figure.

**Comment (5)** Please be consistent with the background of Table 2.

**Answer / **Done, the table now has a consistent white background.

**Comment (6)** Table 3, please add columns of the df and confidence interval. The readers like to see the validity of outcomes.

**Answer / **Confidence interval data have been added to the table, as required.

**Comment (7)** In Table 3, please correct the level of high income (High (>2K)). Also, justify why the income increase of the early symptoms identified is not significant.

**Answer / **Done for the first part (High (> 25K)). In addition, the clarification of this factor was written in the section.

**Comment (8)** In the Discussion, please be consistent in writing the following sentence: “In comparison, previous studies have reported 98.1% among 573 participants in Jordan, 85.4% (n = 5391) to 97.8% (n = 551) in Lebanon, 76.2% among 822 participants in Australia,  76.2% among 609 participants in Iraq, 8.6% among 4,671 in Benin of  West Africa”.

**Answer /** Done, the modifications were made in the manuscript.

**Comment (9)** Please add future work.

**Answer / **The following statement is added to the manuscript (after the conclusion section. “ *Additional studies at the national level could confirm results that are more representative of the general UAE population. More work is needed to raise awareness among people in the UAE through a structured, reliable, evidence-based and accessible health awareness program to target all people in the UAE especially individuals who do not have sufficient knowledge regarding stroke.”*

 Best regards

# Reviewer report: Reviewer response for version 1

**DOI:** 10.5256/f1000research.147377.r204545

**Published:** 2023-09-26

**Authors:** Javedh Shareef

**Affiliations:** 1 Department of Clinical Pharmacy & Pharmacology, RAK College of Pharmacy, RAK Medical & Health Sciences University, Ras Al Khaimah, United Arab Emirates

The authors made a good attempt in carrying out the research related to knowledge and awareness of stroke in the UAE. 

1. Did the author checked the reliability and validity of the study questionnaire?
2. The author has mentioned that the google forms of the questionnaire was distributed through WhatsApp and Facebook. Will there be a possibility of bias if the study participants discuss or share the questions with their friends for right answers. What measures have been taken by the author to avoid this bias?
3. The sample size of the study was estimated to be 384 participants, however we could see that 545 participants were included in the study. Need justification for selecting more number of participants  in the study or else the author need to recalculate the sample size.

Is the work clearly and accurately presented and does it cite the current literature?

Yes

If applicable, is the statistical analysis and its interpretation appropriate?

Yes

Are all the source data underlying the results available to ensure full reproducibility?

Yes

Is the study design appropriate and is the work technically sound?

Yes

Are the conclusions drawn adequately supported by the results?

Yes

Are sufficient details of methods and analysis provided to allow replication by others?

Yes

Reviewer Expertise:

Clinical Pharmacy Practice

I confirm that I have read this submission and believe that I have an appropriate level of expertise to confirm that it is of an acceptable scientific standard, however I have significant reservations, as outlined above.

# Response

**Published:** 2023-10-06

**Authors:** Feras Jirjees

**Affiliations:** University of Sharjah, United Arab Emirates

Dear Reviewer

 Thank you immensely for your insightful comments and recommendations.

**Comment (1)** Did the author checked the reliability and validity of the study questionnaire?

**Answer /** The following statement added to the methods section **:** *The questionnaire was structured similarly to a survey developed and validated in a published study among the general population in Jordan in all aspects that covered knowledge of the stroke with the exception of sociodemographic factors such as economic status due to the discrepancy between the two countries (9). The mentioned survey has been developed using the general principles of good survey design (24)”.* In addition, reliability test was conducted before the study began. The following statement added to the methods section: “ *The reliability of the questionnaires was tested among 12 subjects during the pilot study to detect any difficulties in understanding the topic and meaning of the questions. The Cronbach's alpha value for the items in the second section related to assessing comprehensive knowledge about stroke, stroke risk factors, and stroke consequences was (α = 0.73)*”.

**Comment (2)** The author has mentioned that the google forms of the questionnaire was distributed through WhatsApp and Facebook. Will there be a possibility of bias if the study participants discuss or share the questions with their friends for right answers. What measures have been taken by the author to avoid this bias?

**Answer /** This type of bias is a concern when using this type of data collection tool. Therefore, participants were asked at the beginning of the survey in the “invitation letter” to answer the survey based on their knowledge, without discussing or asking questions related to the survey with anyone, to avoid any relevant bias.

**Comment (3)** The sample size of the study was estimated to be 384 participants, however we could see that 545 participants were included in the study. Need justification for selecting more number of participants  in the study or else the author need to recalculate the sample size.

**Answer / **A larger sample size (n=545) than the minimum requirement (n=384), based on sample size calculation, has been chosen for the current study. This decision is often made to improve the validity of the study. The reasoning behind larger sample size are to increased generalizability as a larger sample size can provide more representative data, and then making it easier to generalize study findings to a broader population. Also, it will improve statistical power with a better chance of detecting real effects or differences if they exist. In addition, it reduced sampling error and enhanced confidence in results to increase the confidence in the study's findings.

 Best regards
